# Impact of 10-Myr scale monsoon dynamics on Mesozoic climate and ecosystems

**DOI:** 10.1038/s41598-020-68542-w

**Published:** 2020-07-23

**Authors:** Masayuki Ikeda, Kazumi Ozaki, Julien Legrand

**Affiliations:** 1 Department of Geosciences, Graduate School of Science, Shizuoka University, Shizuoka, 790-8577 Japan; 2 Department of Earth and Planetary Science, University of Tokyo, Bunkyo, 113-0033 Japan; 3 Department of Environmental Science, Toho University, Chiba, 274-8510 Japan

**Keywords:** Carbon cycle, Element cycles, Stratigraphy, Palaeontology

## Abstract

Earth’s orbital variations on timescales of 10^4^–10^5^ years, known as Milankovitch cycles, have played a critical role in pacing climate change and ecosystem dynamics, through glacial and/or monsoon dynamics. However, the climatic and biotic consequences of these cycles on much longer (~ 10^7^ years) timescales remain unclear, due to a lack of long proxy records with precise age constraints. Here, we show ~ 10-Myr scale variations in early Mesozoic (250–180 Ma) records of lake-level, desert distribution, biogenic-silica burial flux, atmospheric CO_2_ levels (*p*CO_2_), and sea-surface-temperature (SST). Their phase relationships, coupled with carbon cycle modeling results, suggest that orbitally-paced summer monsoon dynamics modulates changes in terrestrial weatherability by ~ 20%, affecting changes in *p*CO_2_ of up to 500–1,000 ppmv and 3–7 °C SST. We also infer that these ~ 10-Myr scale climatic variations could have been causally linked to biotic turnover, size variations in dinosaur footprints, and tetrapod dispersal, potentially through spatio-temporal variations in resource availability and arid-hot climatic barriers at low-middle latitudes.

Quasi-periodic variations in insolation on at least 10^4^ to 10^5^ year orbital-scales (i.e., Milankovitch cycles) are widely accepted as a fundamental pacemaker of the Earth’s surface environments^1,2^. Because amplitudes and frequencies of ~ 2.4-Myr eccentricity cycles and ~ 1.2-Myr obliquity cycles are modulated by inherent chaotic behavior of the Solar System^3,4^, variations in the amplitudes and frequencies of these multi-Myr cycles could also have paced climate changes at longer multi-Myr timescales^5–9^. However, the climatic, biogeochemical, and evolutionary impacts of orbital forcing on multi-Myr timescales are largely unknown.

Ten-Myr scale variations (~ 7**–**13 Myr variations) have been found in carbon isotope data of marine carbonates (δ^13^C_carb_) across at least last 250 Myr, which is interpreted as a result of orbitally-paced monsoon dynamics and related secular changes in climate and carbon transfers^5,7–9^. Although multi-Myr orbital cycles are a potential pacemaker of climatic variations, their theoretically small amplitudes^3, 4^ imply that ~ 10-Myr scale climatic variations would have been amplified through non-linear process(es) in Earth surface system(s)^5–7^. However, to our knowledge, quantitative estimates for ~ 10-Myr scale monsoon dynamics and the impact of this variability on climate related to the carbon cycle have not been explored, primarily because monsoon records longer than several tens of Myr are rare.

Exceptionally well-recorded 10-Myr scale monsoon dynamics are preserved in early Mesozoic sedimentary successions deposited on the supercontinent Pangea and superocean Panthalassa, covering ~ 30 Myr- and ~ 70 Myr-long intervals, respectively^10–13^ (Fig. 1). Precipitation-evaporation cycles recorded on Pangea could have exerted a fundamental control on the rate of terrestrial silicate weathering and dissolved silica (DSi) input into the ocean, which ultimately accumulated as biogenic silica (BSi) in deep sea bedded cherts^13^. Comparing these data with recently developed paleoclimate proxies^14–16^ and fossil records^17–21^ provide a unique opportunity to examine the impact of 10-Myr orbital variations not only on climate dynamics but also on ecosystem dynamics ^6,22^.

**Figure 1:**
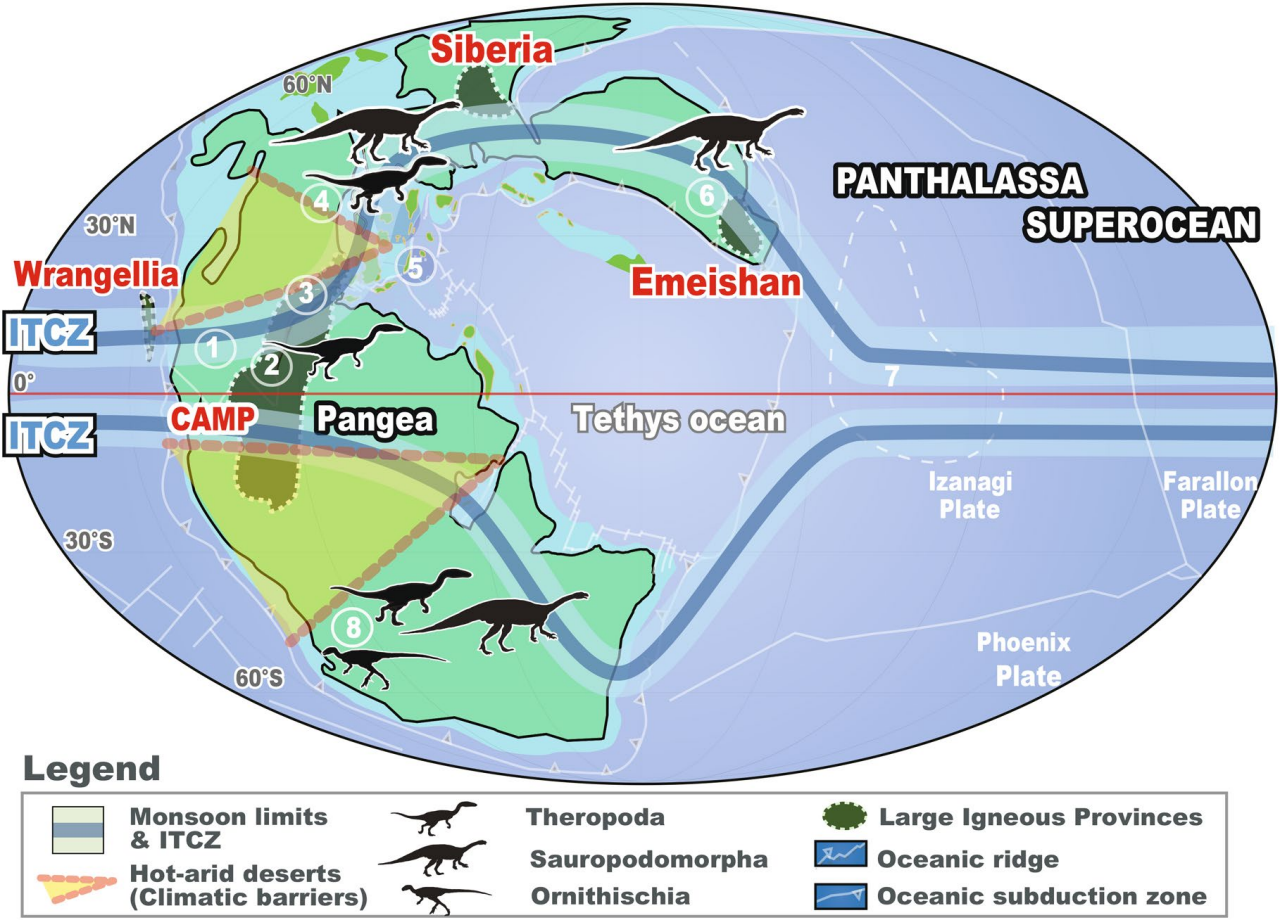
Paleogeography of the Late Triassic world, showing potential distribution of dinosaurs, and localities discussed in this study: **1**, Colorado. **2**, Newark. **3**, Fundy. **4**, Jameson Land. **5**, Pizzo Mondello. **6**, Sichuan. **7**, Inuyama. **8**, Ischigualasto. Palaeogeographic map is modified after ref. 12 and reproduced with permission. The position of summer monsoon limits and inter-tropical convergent zone (ITCZ) at June–July–August (JJA) and December–January–February (DJF) are from geologic records and climate models^13^. The position of extremely hot and arid climatic barriers in the mid-latitudes are inferred in this study.

Here we employ various climate-indicative sedimentological, geochemical, and paleontological records, as well as a geochemical model, to examine the possible impact of multi-Myr orbital variations on climate and ecosystems through monsoon dynamics. Our data consist of (**1**) ~ 30 Myr lake level proxies of the Newark Basin (North America)^11,22,23^, (**2**) ~ 70 Myr terrestrial silicate weathering rate derived from BSi burial flux of pelagic deep-sea bedded chert sequence in the Inuyama area (Japan)^13^, (**3**) spatio-temporal distribution of eolian sediments^24^, (**4**) atmospheric CO_2_ levels (*p*CO_2_) reconstructed from pedogenic carbonates in the Newark–Hartford Basin^14,25,26^, (**5**) sea-surface temperature (SST) reconstructed from oxygen isotope data of conodont apatite (δ^18^O_conodont_) in Tethys ocean^16^, and (**6**) biotic turnover^27–29^, footprint size^19^, and tetrapod dispersal from global paleontological records^19^. Age constraints in our records are provided by high-precision radiometric and astrochronologic ages for the Newark and Inuyama sections, and magnetostratigraphic correlation for other sections (Methods; Supplementary Figure S1). Coupled with these empirical data, we employ a modified version of the GEOCARBSULFvolc model^13^. Together, these unique records provide integrated picture of the impacts of ~ 10-Myr scale orbital variations on global biogeochemical cycles, climate system, and ecosystem.

## Results

###  ~ *10-Myr *scale* monsoon records*

**Figure 2:**
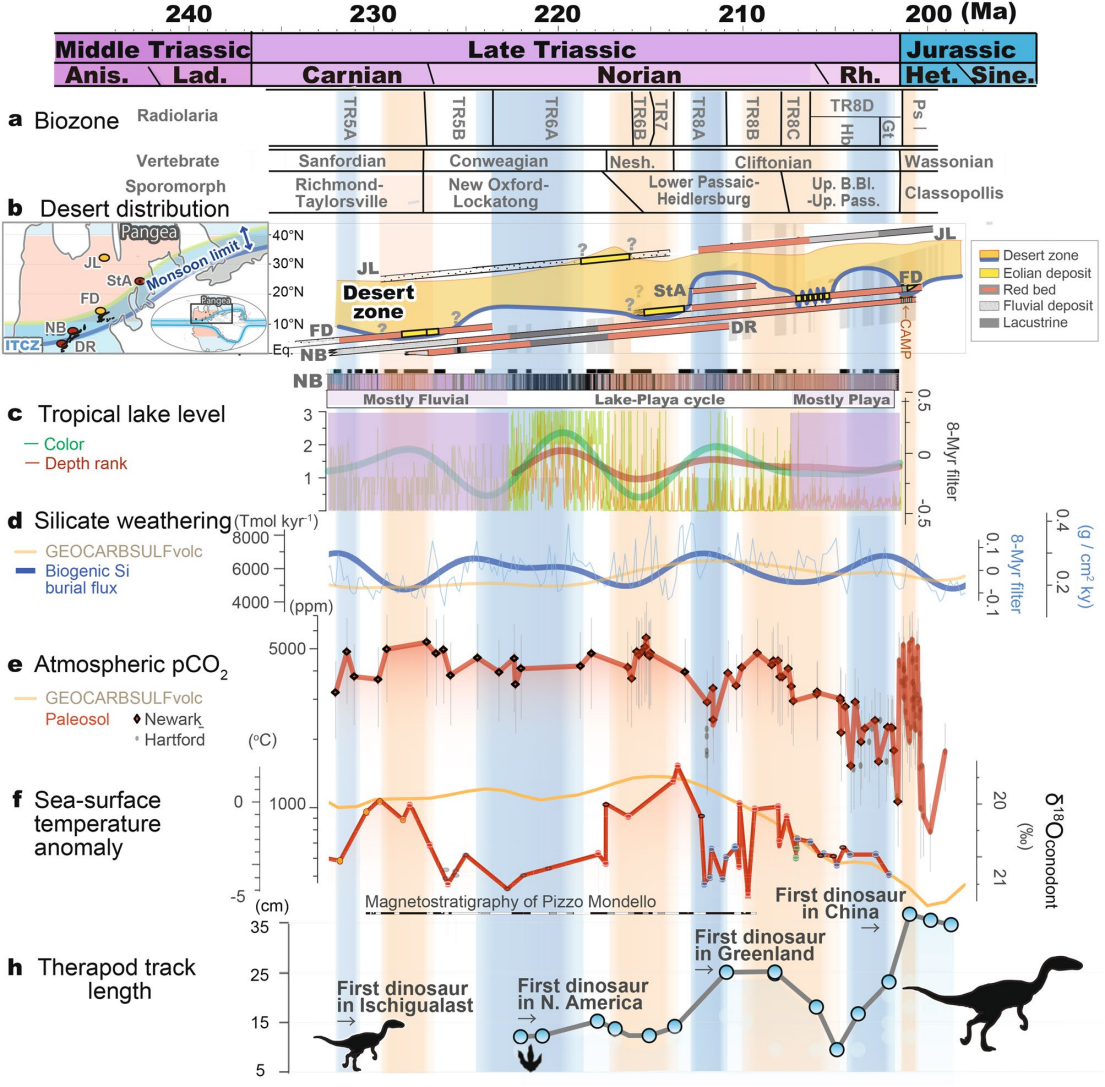
Mesozoic climate-indicative sedimentological and geochemical records. (**a**) Biostratigraphy of the Newark and Inuyama areas^27–29^. (**b**) Stratigraphic chart of eolian strata in low-latitude Pangea^32^. (**c**) Tropical lake level changes in the Newark Basin (**d**) Silicate weathering rate from biogenic silica (BSi) burial flux of deep-sea Inuyama section and the GEOCARBSULFvolc result^13^. (**e**) Atmospheric *p*CO_2_ records from pedogenic carbonate in the Newark and Hartford basins^14,25,26^ and the GEOCARBSULFvolc result^13^. (**f**) Sea-surface temperature reconstructed from conodont apatite^15,16^. (**g**) Maximum size of theropod footprints with first appearance of dinosaurs in Ischigualast, North America, Greenland, and China^12,17,19,21^. Details on age uncertainties for these data are given in Supplementary Figure S1. *Anis.* Anisian, *Lad.* Ladinian, *Rh.* Rhaetian, *Het.* Hettangian, *FD* Fundy Basin, *NB* Newark Basin, *DR* Deep River Basin, *StA* Saint Audrie’s Bay, *JL* Jameson Land.

**Figure 3:**
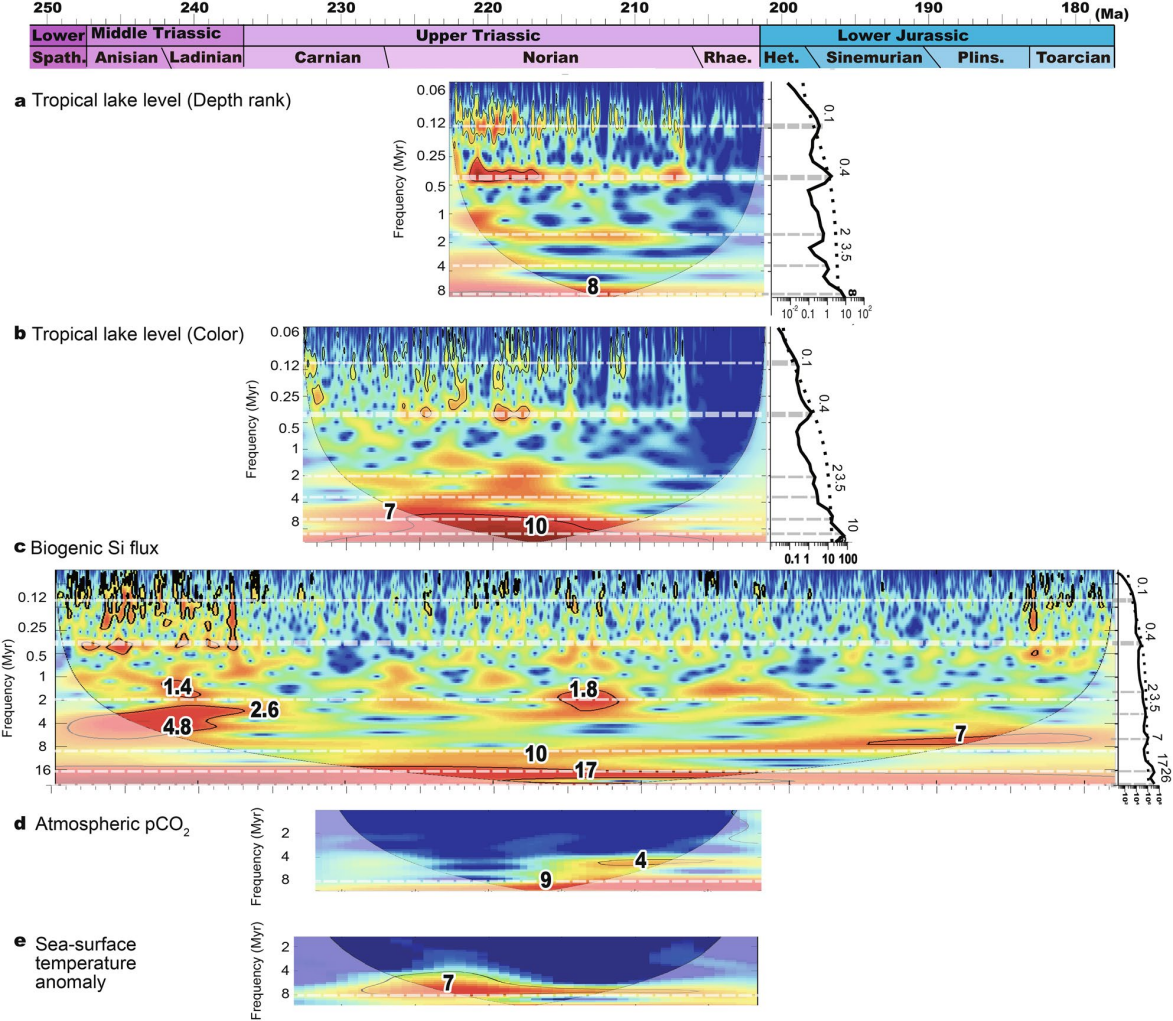
Wavelet spectra of depth rank and color of Newark Basin, biogenic Si flux of Inuyama chert, *p*CO_2_ of Newark–Hartford paleosols, and sea surface temperature (SST) of Tethyan conodont apatite records. Details of age uncertainties of each data are given in Supplementary Figure S1. *Spath.* Spathian, *Het.* He.

During the Late Triassic, lake level and atmospheric *p*CO_2_ records from the Newark Basin show slightly decreasing trends, whereas the Inuyama BSi burial flux data from superocean Panthalassa and SST data from δ^18^O_conodont_ in the Tethys show negligible trends (Fig. 2c–f). The ages of eolian deposits in the Fundy Basin are ~ 230 Ma, ~ 215–213.5 Ma, 207.2–204.6 Ma, and 201.5 Ma, based on biostratigraphy for Carnian to 215 Ma, and magneto-cyclostratigraphic correlation with the Newark Basin for 215–201.5 Ma and U–Pb age of 201.566 ± 0.031 Ma for the North Mountain Basalt just below the fluvio-eolian McCoy Brook Formation^11,30^ (Fig. 2b; Supplementary Figure S1). Time-series analysis of lake level data from the Newark Basin shows cycles with periods of approximately 0.1, 0.4, 8, and 10 Myr cycle above 90% confidence level with secondary peaks at 2 and 3.5 Myr cycle^23,31^ (Fig. 3). Although ~ 8- to 10-Myr cycle is close to the Nyquist frequency of these lake level data, their variances are high during the Late Triassic (Figs. 2 and 3). Time-series analysis of the Inuyama BSi burial flux data shows cycles with periods of approximately 0.1, 0.4, 7, and 17 Myr^13^ (Fig. 3). Wavelet spectra of these data also show frequency modulation of approximately 10 Myr cycles (between 7 and 11 Myr), which is similar to that of amplitude modulation of ~ 2 Myr eccentricity cycle^3,4,6^. In the present study, we focus on the prominent cycles of ~ 10 Myr (7–13 Myr) duration in these two records, and their possible link with the paleoclimatic and paleontological records introduced above. Time-series analysis of atmospheric *p*CO_2_ data of the Newark Basin shows cycles with periods of approximately 4 and 9 Myr^13^ (Fig. 3). Time-series analysis of SST data shows cycles with periods of approximately 7 Myr^13^ (Fig. 3).

Based on the high-resolution astrochronologic frameworks, in conjunction with U–Pb radiometric ages correlated by chemo-bio-magnetostratigraphy and an ejecta layer of Manicouagan origin^10–12,33,34^ (See Methods; Supplementary Figure S1), the 10-Myr scale changes in lake level and BSi burial flux show a nearly in-phase relationship during a period of cyclic lake-playa development in the Lockatong and lower Passaic formations of the Newark Basin. In contrast, the 10-Myr scale changes in lake level and BSi burial flux are not in-phase during a period of mostly fluvial sedimentation in the Newark Basin (Stockton Formation), and the mostly playa environment of the upper Passaic Formation (Fig. 2b,c). Overall, the BSi burial flux data are out-of-phase with ~ 10-Myr scale changes in *p*CO_2_ of up to ~ 1,000 ppmv, and SST up to ~ 7°C^14,15^ (Fig. 2c,d).

**Figure 4:**
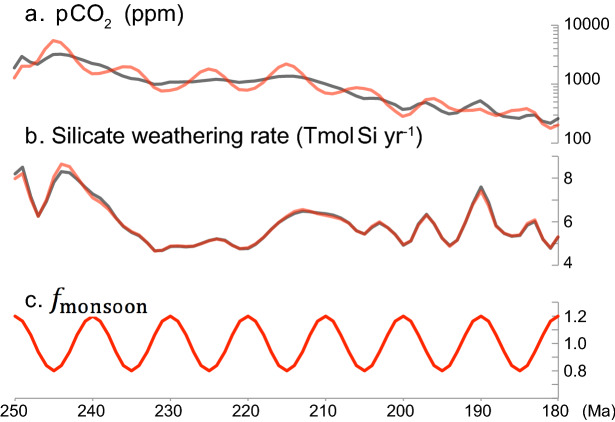
Model results showing the effect of terrestrial weatherability on atmospheric *p*CO_2_ and silicate weathering rate. (**a**) Atmospheric *p*CO_2_ concentration. (**b**) Global silicate weathering rate. (**c**) *f*_monsoon_ (a linear factor for terrestrial weatherability in the revised GEOCARBSULFvolc model; Methods). Black lines show our control run and red lines show 20% changes in weatherability, as represented in *f*_monsoon_.

Based on a sensitivity experiment in a new weatherability parameter *f*_monsoon_, which scales terrestrial weatherability in the revised GEOCARBSULFvolc model^13^, we found that variations of just 20% in *f*_monsoon_ could yield *p*CO_2_ variations as large as 500–1,000 ppmv, with a negligible impact on global silicate weathering rate (Fig. 4). These *p*CO_2_ variations are consistent with the proxy records^14,15^.

## Discussion

###  ~ 10-Myr scale environmental changes amplified through biogeochemical cycle

Precession- and eccentricity-related orbital signals (~ 20-kyr to ~ 2 Myr) have been recorded previously in lake level data from the Newark Basin, deposited in equatorial Pangea, and in BSi burial flux from Inuyama, deposited in low-latitude Panthalassa^10,13,22,23^. Here, we now also recognize ~ 10-Myr (7 to 13 Myr) scale changes in lake level, BSi burial flux, atmospheric *p*CO_2_, and SST records with unstable frequency (Fig. 3). Their in-phase relationship in the most environmentally quiescent and thus stratigraphically reliable lacustrine interval in the Newark Basin (See *Age model*) supports their orbital origin, because amplitudes of ~ 2 Myr to 100 kyr eccentricity and ~ 21 kyr precession cycles are also modulated on ~ 10-Myr timescale with changes in frequency between 7–13 Myr, according to astronomical theory^4^.

The out-of-phase relationship between BSi and *p*CO_2_/SST suggests that variations in CO_2_ degassing rate would not be a major driver of carbon cycle during this interval, because variations of degassing rate predicts a coupled evolution of atmospheric *p*CO_2_ and terrestrial silicate weathering rate on this timescale (Fig. 2c–e). This idea is consistent with no Large Igneous Province (LIP) activity across most of the Late Triassic. An alternative explanation is variations in terrestrial weatherability. Our modeling demonstrates that 10–20% variations in global weatherability could yield *p*CO_2_ variations as large as 500–1,000 ppmv, with a minor impact on global silicate weathering rate (Fig. 3). We thus suggest that the decoupling between *p*CO_2_ and global silicate weathering rate can be explained if terrestrial weatherability was controlled by orbitally-paced mega-monsoon dynamics at least during the Late Triassic.

A possible factor changing terrestrial weatherability would be orbital-scale latitudinal variations in summer monsoon limits around highly-weatherable volcanic rocks, such as those contained within volcanic arcs, the Siberian traps, Emeishan basalts, and Wrangellia basalts^13^ (Fig. 1). At the present day, volcanic rocks exposed to a monsoonal climate occupy only ~ 10% of land areas, but they are responsible for more than 70% of DSi fluxes to the ocean^35^. Therefore, up to 20% variations in terrestrial weatherability could be partly explained by small changes in such highly-weatherable areas. During the early Mesozoic, the presence of the supercontinent Pangea would have enhanced monsoonal circulation, creating a so-called ‘mega-monsoon^36^′, driven by the large contrast in heat capacity between this supercontinent and the superocean Panthalassa (Fig. 1). Such strong mega-monsoonal circulation would have further enhanced this areal fraction through the larger spatial changes in the monsoonal regions (Fig. 1)^36,37^.

This idea is supported by ~ 10-Myr scale reappearance of the eolian succession in the Fundy Basin, Canada (~ 10–20°N) during the Late Triassic during the periods of low BSi burial in Panthalassa during ~ 230 Ma, ~ 215–213.5 Ma, 207.2–204.6 Ma, and 201.5 Ma^11^ (Fig. 2b). Such ~ 10-Myr scale latitudinal shifts of desert distribution during these intensified summer monsoon periods could have enhanced weatherability by a factor significantly relative to extremely arid periods^13^.

Taken together, we suggest that the processes discussed above could have amplified ~ 10-Myr scale orbitally forced changes in terrestrial weatherability of up to 10–20% (Fig. 3). We also acknowledge that orbitally-forced monsoon dynamics may not be the sole cause of the observed *p*CO_2_ and temperature variations, and other unidentified processes, such as the changes in topography and imbalance of organic carbon subcycle, may contribute. Northward drift of Pangea and uplift of Cordilleran magmatic arc complex could have caused changes in weatherability^38,39^, but they are difficult to explain on 10-Myr scale changes. The *p*CO_2_ changes driven by the changes in organic carbon burial are partly supported by δ^13^C_carb_ records with ~ 1‰, but they had only a negligible impact during the Late Triassic (~ 100 ppmv)^13^. Degassing rate might also have changed on 10-Myr timescale, but it is difficult to constrain and there is no LIP activity across the most of Late Triassic. Although further quantitative examination is required, decoupled secular trends in silicate weathering (and BSi) burial flux with *p*CO_2_/SST during the Late Triassic suggest that long-term monsoon-driven weatherability changes (see Methods) are one of the primary drivers of the *p*CO_2_ and SST at least during the Late Triassic.

### Potential impacts on ecosystem dynamics

**Figure 5:**
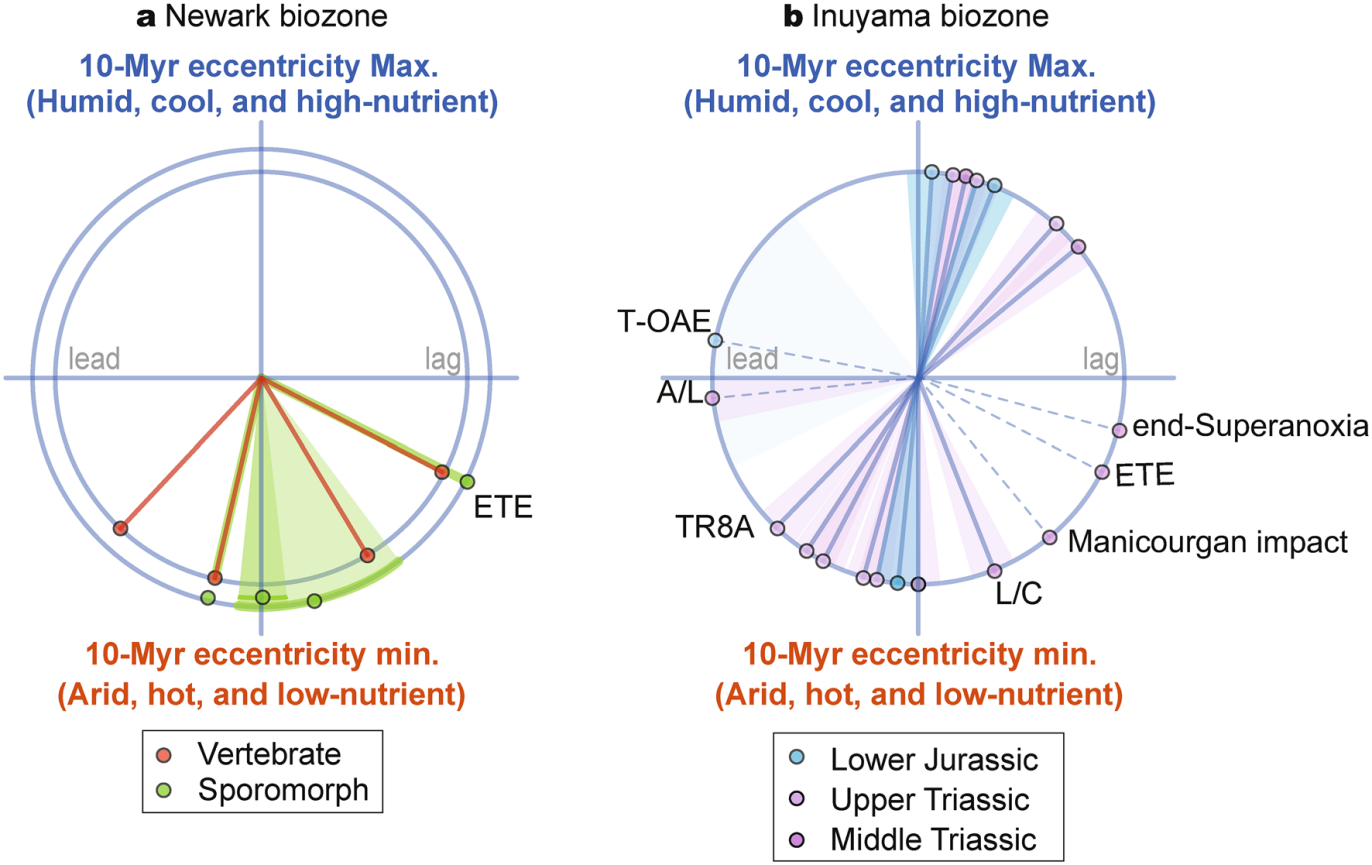
Phase wheel showing the timing of biozone boundaries^27–29^ relative to ~ 10-Myr eccentricity cycles based on the BSi burial flux records (Fig. 2). Phase lags increase in clockwise direction (3 o’clock equals 90° or 2.5 Myr phase lag). Shaded areas represent uncertainties of the zone boundaries. A/L = Anisian/Ladinian, L/C = Ladinian/Carnian, ETE = End-Triassic Extinction, T-OAE = Toarcian Oceanic Anoxic Event.

The ~ 10-Myr scale climatic variations inferred from geologic records that we observe have potentially important ramifications for the evolution of terrestrial-marine ecosystems during the Triassic. Vertebrate and floral zone boundaries in the Newark Basin occur coevally with low silicate weathering and high *p*CO_2_/hot climate^27,28^ (Fig. 2a–f). Radiolarian zone boundaries in the pelagic Panthalassa are not only within ± 1 Myr of the ~ 10-Myr scale lowest eccentricity periods, but also within ± 1 Myr of the ~ 10-Myr scale highest eccentricity periods^29^ (Figs. 2 and 5). Because amplitudes of ~ 2 Myr to 100 kyr eccentricity and 20 kyr precession cycles are also modulated by ~ 10-Myr cycles, these biotic turnovers could have been paced with low/high variability of insolation and summer monsoon intensity and/or associated ~ 10-Myr scale climate change (Fig. 2a–g).

Similar Myr-scale orbital pacing of biotic turnovers has been reported from Neogene mammal records, suggesting that turnovers occurred during minima of 2.4-Myr eccentricity cycles and nodes of 1.2-Myr obliquity cycle, due to cooling and aridification^40^. Contrary to the Neogene icehouse, most biotic turnovers during the Triassic hothouse interval occurred during ~ 10-Myr scale eccentricity minima associated with lethally hot and arid periods (Fig. 2a–f). Such 10-Myr scale climate changes are consistent with floral changes^27^. On the other hand, 10-Myr scale eccentricity maxima led to cool and humid climate conditions during the Triassic. This would not impact on terrestrial ecosystems, but such conditions might have affected radiolaria through increased nutrient flux and/or subsequent variations in radiolarian distribution (Fig. 2g). Therefore, ~ 10-Myr scale monsoon dynamics could have also paced biotic turnovers, at least during the Triassic (Fig. 2).

Such ~ 10-Myr scale extreme climate variations might have allowed terrestrial faunal dispersal, including that of dinosaurs, by reducing extremely arid and hot climatic barriers in mid-latitude regions (Figs. 1, 2h)^41^. Intensified summer monsoon activity, associated with humidification, shrinkage of desert areas, and global cooling, could have lowered the extremely arid and hot climatic barriers (Fig. 2a–e). These factors might have facilitated the dispersal of tetrapods over the climatic barriers in the northern mid-latitudes, at least during the mid-Norian (~ 212 ± 2 Ma), based on the first appearance of theropod dinosaurs in the Jamesen Land, Greenland constrained by magneto-astrochronology^42^ (Fig. 2g; Supplementary Figure S1). Similar tetrapod dispersal might have occurred during 10-Myr scale eccentricity maxima, such as at the Olenekian-Anisian for *Arizonasaurus* (~ 247 Ma)^43^, mid-Carnian for phytosaurs (~ 232 Ma; Carnian Pluvial Event)^20^, and early Norian for dinosaurs and mammaliaformes (~ 222 Ma)^17, 18^, and middle Rhaetian for dinosaurs (~ 204 Ma)^21^, in addition to the mid-Norian, possibly through similar mechanisms. Nevertheless, the geologic data are limited and subject to large age uncertainties for the first appearance of fossils (see Methods and Supplementary Figure S1).

The probable timing of dinosaur dispersal ~ 212 ± 2 Ma might be coincident with an increase in maximum size of theropod dinosaur footprints from ~ 15 cm to ~ 25 cm in the Newark Supergroup^19^ (Fig. 2h). This increase in the size of theropod footprints could be explained by dispersal from some unknown locations and/or by an ecological/evolutionary event in this region^19^. The former possibility is consistent with the probable timing of dinosaur dispersal (Fig. 2h). Additionally, given the paleoclimate records shown, such gigantism under cool and humid climate conditions at ~ 212 ± 2 Ma could have been supported by increased availability of water and food resources. Resource availability is positively correlated with migration distance for modern terrestrial mammals and reptiles^44,45^, implying that resource availability might have been a factor in hemisphere-scale long distance dispersal of dinosaurs during the Late Triassic.

**Figure 6:**
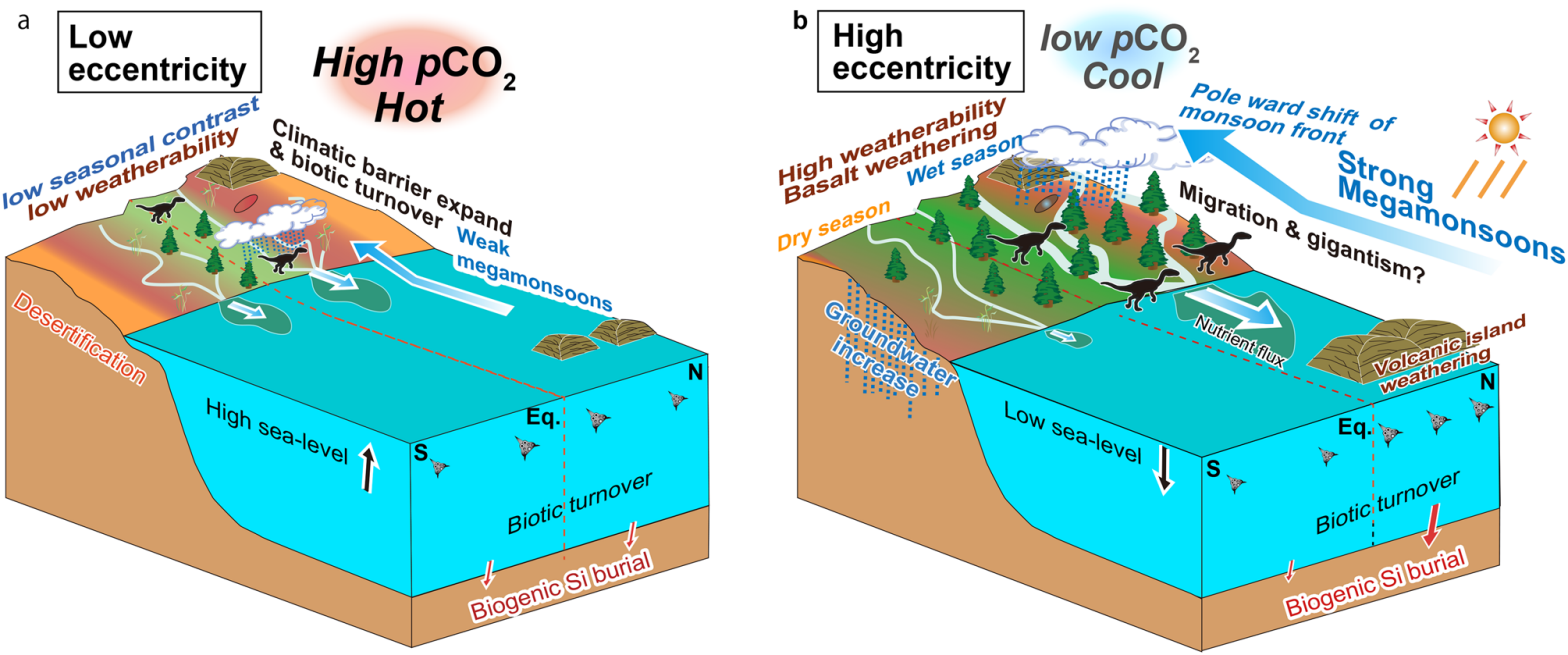
Schematic cartoon of paleoenvironmental changes linked to minima (**a**) and maxima (**b**) of ~ 10-Myr eccentricity. In this model, the carbon cycle is highly influenced by the seasonal dynamics of hydrological processes and continental weathering. Dinosaur dispersal from equator to northern mid-latitude could have occurred at the mid-Norian (~ 212 ± 2 Ma).

The postulated astronomical hypothesis for ~ 10-Myr-scale variations in climate and ecosystems provides a crucial missing piece in the puzzle of Earth system dynamics on this timescale, and provides a probable mechanism to explain changes in monsoon dynamics, desert distribution, sea-level, atmospheric CO_2_ levels, SST, and biotic turnover at least during the Late Triassic (Fig. 6). Because ~ 10-Myr scale orbital cycles would have been operative throughout Earth history^4^, further investigation on the impact of ~ 10-Myr scale orbital cycles on Earth’s climate and ecosystems may help explain similar processes in other time periods.

## Methods

### Age models

To establish numerical age models for sedimentological, geochemical and paleontological records, we mainly used biostratigraphy-independent age constraints, such as radioisotopic and astrochronologic ages, in conjunction with magneto-chemostratigraphy (Supplementary Figure S1). Astrochronologic age model for the Newark lacustrine sequence is constructed based on lake-level cyclostratigraphy, which is supported by detailed zircon U–Pb ages from the same succession and other sections correlated by magnetostratigraphy^11,12^. The astrochronomically-tuned magnetic polarity time scale (APTS) of the Newark–Hartford section is anchored by U–Pb ages of intercalated flood basalts^30^, and is consistent with 20 zircon U–Pb ages of other sections correlated by magnetostratigraphy^11,34^. The astrochronology of the basal fluvial interval (Stockton Formation) was calibrated by assumed 405 kyr cycle despite a lack of higher frequency cycles, and was extrapolated assuming constant sedimentation rate^23^. Although precession and eccentricity signals in the mostly playa interval (upper Passaic Formation) are muted, the chronology of this interval was validated by projected U–Pb dates into Newark from the Colorado Plateau by magnetostratigraphy^34^.

The astrochronology of the Inuyama deep-sea sequence is anchored at the end-Triassic radiolarian extinction interval^46^ (201.5 ± 0.2 Ma^47^), which is constrained by U–Pb ages of the Pucara section (Peru)^48^. The Inuyama astronomical time scale (Inuyama-ATS) is supported by 21 zircon U–Pb ages from other sections correlated by radiolarian-conodont biostratigraphy, carbon isotope stratigraphy, and an ejecta layer from the Manicouagan crater^10,33^. After Ikeda and Tada^10^, occurrence of a base-Rhaetian marker radiolaria, *Betracurum deweveri,* between ~ 212 and 209.5 ± 0.2 Ma is consistent with that in the Pianola-Abriola section calibrated by Newark-ATPS^49^ (Supplementary Figure 1S). Astrochronologic age of 183.25–183.01 ± 0.2 Ma for the Pliensbachian/Toarcian boundary based on carbon isotope stratigraphy in the Inuyama area^33^ is also consistent with U–Pb ages of this boundary^50^ and the onset of Karoo–Ferrar volcanism^51^. Ages for other marine and non-marine sections are compiled based on U–Pb ages and bio-chemo-magnetostratigraphic correlation, which have uncertainties of up to several Myr according to magnetostratigraphic correlation to the Newark–Hartford APTS^49^ (Supplementary Figure S1).

### Data preparation

We compiled climate-indicative sedimentological and geochemical records, which are potentially related with multi-Myr orbitally-paced climate and ecosystem variability. Depth rank, a proxy of relative lake depth, is a classification of facies by suites of sedimentary structures in which facies are assigned a value of 0 to 5 in order of increasing relative water depth (7, 17). Color is related to the reduction–oxidation state and organic carbon contents in the sedimentary rocks. Semi-quantitative ranks of color are assigned a value of 0 to 5 in order of increasing relative water depth (7, 17). The sensitivity of the lake level proxies to summer monsoon intensity could be low during periods of the mostly fluvial Stockton Formation and the mostly playa interval of the upper Passaic Formation in the Newark Basin, due to the low lake-level caused by basin tectonics and persistent aridification by northward movement of Pangea, respectively^22^.

The BSi burial flux of deep-sea chert was calculated from a chert bed thickness dataset in the Inuyama area^10^ based on its positive correlation with the BSi amounts for one chert-shale couplet^13^ and astrochronology above^10^. We here also consider the diagenetic segregation, which migrated BSi from the layers with low BSi content to adjacent layers with high BSi content, as a mechanism for amplification of precession-scale changes in BSi content. The estimated average value of BSi burial flux is ~ 90% of the modern value, suggesting that BSi in the bedded chert was a major sink for oceanic dissolved silica (DSi) and globally representative. Thus, over timescales longer than the residence time of oceanic DSi (< ~ 100 kyr^52^), BSi burial flux as bedded chert should be proportional to global DSi input flux mainly through global chemical weathering^13^. Because terrestrial chemical weathering rates can be approximated as a product of linear precipitation and Arrhenius temperature functions, the observed changes in the BSi burial flux could have been controlled by summer monsoon intensity on orbital timescales^13,53^. This idea is supported by similar secular pattern of global silicate weathering rate calculated by the revised GEOCARBSULFvolc model^13,53^.

Proxies for desert distribution and *p*CO_2_ are mostly compiled from eolian sediments and pedogenic carbonate, respectively, in the rift basins of the Central Atlantic Margin, such as the Newark Basin, due to their well-constrained paleolatitudes and age models^11,14^. SST data is compiled from δ^18^O_conodont_^16^ in shallow marine successions of the Tethys with age model adapted to the revised numerical age model^15^ (Supplementary Figure S1).

### Time series analysis

The performed time series analysis used a series of Matlab algorithms modified from those developed by Torrence and Compo (all filtered, detrended, normalized, and padded in the same way)^54^. This software can identify whether peaks in the spectrum of time series are significant against a red-noise (autoregressive lag1) background spectrum.

### Modified GEOCARBSULFvolc model and ***f***_monsoon_

We employed the long-term carbon and sulfur geochemical model, GEOCARBSULFvolc^13, 55^, to examine the Myr-scale global biogeochemical cycle dynamics. We assumed Royer et al.^55^′s initial condition at *t* = 570 Ma (where *t* is age) and ran the model with their input arrays until *t* = 250 Ma. For our target interval (*t* = 250–180 Ma), we used a previously compiled isotope series of carbon, sulfur and strontium, which enable us to obtain a multimillion-year timescale global silicate weathering rate^13^. The time step was set at 1 Myr. Other parameter values are set at the standard value of Royer et al.^55^ (listed in Table S3 of Ikeda et al.^13^).

In this study, we introduce a single parameter, *f*_monsoon_, to quantify the hypothetical impact of monsoonal dynamics on terrestrial weatherability of silicate rocks (*f*_monsoon_ = 1 for the standard run presented in Fig. 2):

1 $$F_{\text{wsi}} = f_{\text{monsoon}} \left( t \right)f_{\text{volc}} \left( t \right)f_{\text{Bt}} \left( {{\text{CO}}_{2} } \right)f_{\text{Bb}} \left( {{\text{CO}}_{2} } \right)f_{\text{R}} \left( t \right)f_{{\text{E}}} \left( t \right)\left( {f_{\text{Aw}} f_{\text{D}}}\right)^{0.65} F_{\text{wsi}} \left( 0 \right)$$

where *F*_wsi_ denotes the global silicate weathering flux. We performed a sensitivity experiment in which *f*_monsoon_ was forced in the range of 0.8–1.2 with a 10 Myr periodicity (Fig. 3):

2 $$f_{{{\text{monsoon}}}} \left( t \right) = 1 + 0.2\sin \left( {2\pi \frac{t + 255}{{10}}} \right)$$

In the GEOCARBSULF model, global river runoff (*f*_D_) and fraction of land area undergoing chemical weathering (*f*_Aw_/*f*_A_) are critical factors for atmospheric *p*CO_2_ variation related with monsoon dynamics. According to the uncertainty analysis of this model, however, their uncertainties of approximately 20% are not significant for *p*CO_2_ estimates^55^. Other factors, such as climate sensitivity and the ratio of chemical weathering by gymnosperms to angiosperms (GYM) have larger uncertainties for *p*CO_2_ estimate^55^. Nevertheless, climate sensitivity is relatively stable throughout the Late Triassic according to the consistent relation between atmospheric *p*CO_2_ and SST in geologic records^15^. Effect of GYM should also be negligible due to the rise of angiosperms during the Cretaceous^56^.

Contrary to these uncertainties examined^55^, ~ 10-Myr scale variations in *f*_Aw_/*f*_A_, and possibly *f*_D_ could be much larger based on the monsoon-related records, including lake-level, desert distribution and BSi burial flux (Fig. 2b,c,d). On the other hand, considering the extremely hot climate with high *p*CO_2_ conditions^15^, the effect of temperature might not be the limiting factor controlling silicate weathering. Although further quantitative estimates are critical to an improved understanding of our hypothesis, 10-Myr scale monsoon dynamics could have affected terrestrial weatherability and carbon cycle dynamics.

The effect of *f*_monsoon_ on the global silicate weathering rate is negligible, because global silicate weathering rate should be proportional to the imbalance of the carbon cycle (e.g. degassing rate and organic carbon burial/weathering) on Myr timescales (Fig. 3). Ten-Myr scale changes in degassing rate might be a potential candidate to explain geological *p*CO_2_ records, although it is difficult to constrain degassing rates on this timescale and there are no major LIPs during the Late Triassic. Additionally, 10-Myr scale imbalances of the organic carbon cycle with high organic carbon burial during low eccentricity periods, as recorded in δ^13^C_carb_ records^5, 7^, could have modulated the 10-Myr cycle in global silicate weathering flux, but have a negligible effect on *p*CO_2_ (Fig. 3). Further high-resolution δ^13^C_carb_ records could confirm the contribution of organic carbon cycle on silicate weathering.

### Fossil records

To estimate the influence of 10-Myr scale climate changes on ecosystems, we used fossil records with biostratigraphy-independent age constraints, such as radioisotopic dates and magneto-astrochronology, to avoid circularity in dating of paleobiogeographic events by biostratigraphy (Supplementary Figure S1). The ages of biozone boundaries are estimated by astrochronology, as described above. To assess the timing of dispersal events, we compiled the time-calibrated phylogeny of tetrapods, including taxa without biostratigraphy-independent age constraints, in Supplementary Figure S1. Although some works have pointed to a possible northern hemisphere origin of dinosaurs^57^, most Triassic dinosaur records in the middle latitudes of the northern hemisphere lack biostratigraphy-independent age constraints, except for those in Jameson Land, Greenland, which have cyclo-magnetostratigraphic age constraints (Supplementary Figure S1). Another Late Triassic dinosaur record is sauropodomorph footprints in the Rhaetian Xujiahe Formation in China, dated as ~ 204 Ma based on magneto-cylostratigraphic correlation with Newark-ATPS^21,58^, which age is consistent with disappearance of eolian deposits^24^ and increased size of theropod footprints in the Newark Supergroup^19^.

## Supplementary information

Supplementary file1 (DOCX 2369 kb)
