# Supplementary material for: Impact of 10-Myr scale monsoon dynamics on Mesozoic climate and ecosystems
Source: Sci Rep. 2020 Jul 23;10:11984. doi: 10.1038/s41598-020-68542-w (PMC7378230; doi:10.1038/s41598-020-68542-w)
Supplement: Supplementary file 1 — Supplementary file1 (DOCX 2369 kb) [file 41598_2020_68542_MOESM1_ESM.docx]

sedimentary and fossil records.

**Impact of 10-Myr scale monsoon dynamics on Mesozoic climate and ecosystems**

Masayuki Ikeda^a,b^*, Kazumi Ozaki^c^, and Legrand Julien^a,b^

^a^ Department of Geosciences, Graduate School of Science, Shizuoka University, Shizuoka, 790-8577, Japan.

^b^ Department of Earth and Planetary Science, University of Tokyo, Bunkyo, 113-0033, Japan

^c^ Department of Environmental Science, Toho University, Chiba, 274-8510, Japan

This Supplementary Information contains Figures S1 which shows chronostratigraphic correlation amongst the early Mesozoic sequences based mainly on biostratigraphy-independent age models with some biostratigraphic records.


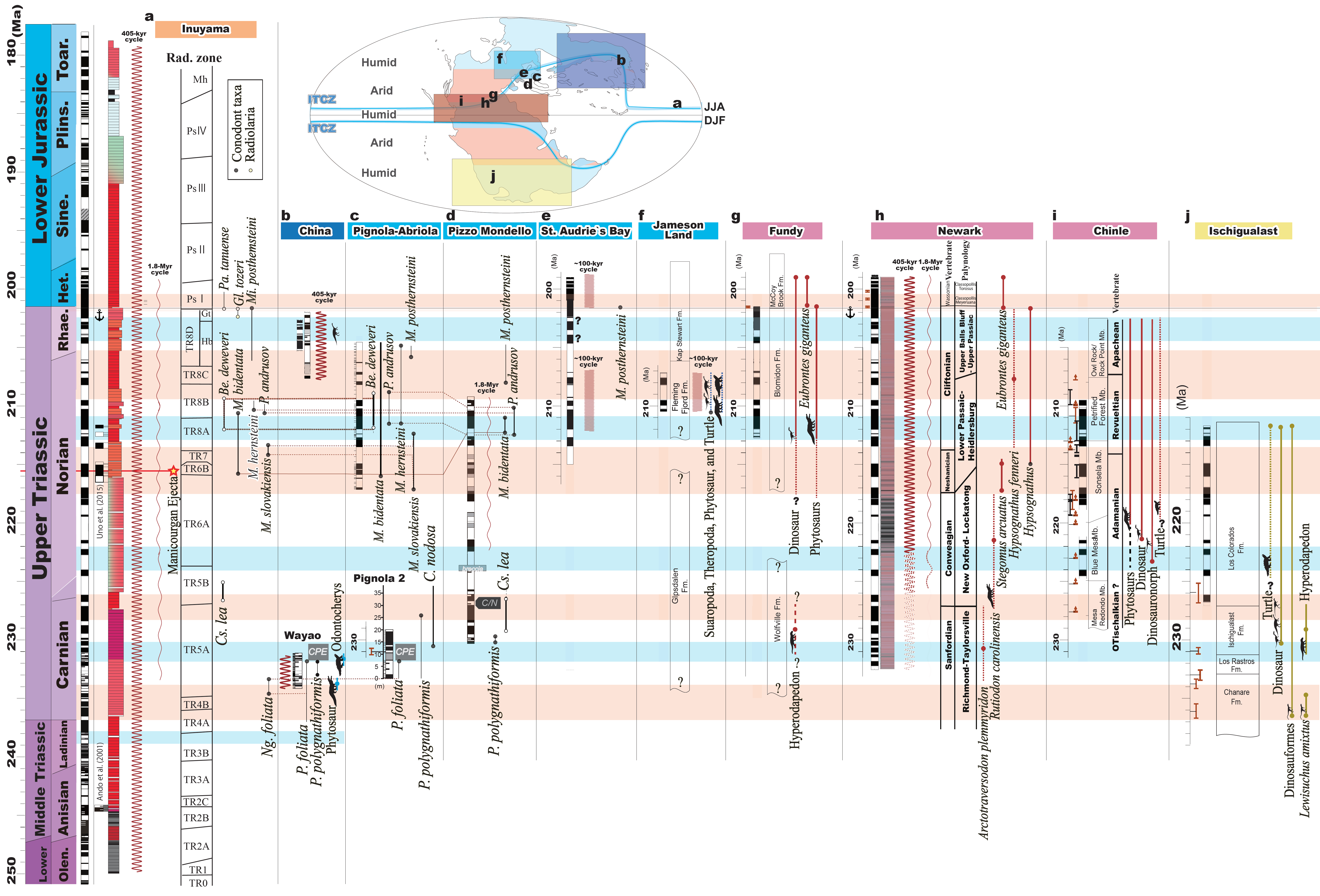
**Supplementary Figure S1.** Chronostratigraphic correlation between early Mesozoic sequences. **a**, Inuyama ( Sugiyama, 1997; Ikeda and Tada, 2014; Onoue et al., 2016; Yamashita et al., 2018). **b**, Wayao section (China; Minzoni et al., 2015; Zhang et al., 2017), **c**, Pignola-Arbiola (Maron et al., 2015), **d**, Pizzo Mondello (Muttoni et al., 2004; Muttoni et al., 2014; Onoue et al., 2018). **e**, St. Audrie’s Bay (Hounslow et al., 2004; Kemp and Coe, 2007; Ruhl et al., 2010). **f**, Jameson Land, Greenland (Kent and Clemmensen, 1996; Lallensack et al., 2017). **g**, Fundy, Canada (Kent and Olsen, 2000; Sues and Olsen, 2015). **h**, Newark, USA (Cornet, 1985; Huber et al., 1993; Kent et al., 2017; Olsen and Kent, 1996; Olsen and Kent, 1999; Olsen et al., 2002; Olsen et al., 2011). **i,** Chinle, USA (Kent et al., 2018; Ramezani et al., 2014; Ramezani et al., 2011). **j**, Ischigualast (Kent et al., 2014; Ezcurra et al., 2017). Numerical age models are based on U-Pb ages in conjunction with astrochronology. Magnetostratigraphic correlations are based on the original papers. Inset map is a Pangean paleogeography showing the locations. The orange and blue shaded areas represent the 10-Myr scale maxima/minima of BSi burial flux.

**References**

Cornet, B., Olsen, P.E., 1985. A summary of the biostratigraphy of the Newark Supergroup of eastern North America with comments on Early Mesozoic provinciality. In: Weber, R. (Ed.), Simposio Sobre Floras del Triasico Tardio, su Fitogeografia y Paleoecologia: Memoria, III Congresso Latinoamericano de Paleontologia, Mexico. Instituto de Geologia Universidad Nacional Autonoma de Mexico, Mexico City., 67–81.

Diedrich, C., 2015. Isochirotherium trackways, their possible trackmakers (? Arizonasaurus): intercontinental giant archosaur migrations in the Middle Triassic tsunami-influenced carbonate intertidal mud flats of the European Germanic Basin. Carbonates and Evaporites 30, 229-252.

Ezcurra, M.D., Fiorelli, L.E., Martinelli, A.G., Rocher, S., von Baczko, M.B., Ezpeleta, M., Taborda, J.R., Hechenleitner, E.M., Trotteyn, M.J., Desojo, J.B., 2017. Deep faunistic turnovers preceded the rise of dinosaurs in southwestern Pangaea. Nature Ecology & Evolution, 1.

Hounslow, M.W., Posen, P.E., Warrington, G., 2004. Magnetostratigraphy and biostratigraphy of the Upper Triassic and lowermost Jurassic succession, St. Audrie's Bay, UK. Palaeogeography, Palaeoclimatology, Palaeoecology 213, 331-358.

Huber, P., Lucas, S.G., Hunt, A.P., 1993. Vertebrate biochronology of the Newark Supergroup Triassic, eastern North America. 3, 179–186. N. M. Mus. Nat. Hist. Sci. Bull. 3, 179-186.

Ikeda, M., Tada, R., 2014. A 70 million year astronomical time scale for the deep-sea bedded chert sequence (Inuyama, Japan): Implications for Triassic–Jurassic geochronology. Earth and Planetary Science Letters 399, 30-43.

Ikeda, M. & Tada, R., 2020. Reconstruction of the chaotic behavior of the Solar System from geologic records. *Earth and Planetary Science Letters* **537**, 116-168.

Ikeda, M., Tada, R., Ozaki, K., 2017. Astronomical pacing of the global silica cycle recorded in Mesozoic bedded cherts. Nature communications 8, 15532.

Joyce, W.G., 2017. A review of the fossil record of basal Mesozoic turtles. Bulletin of the Peabody Museum of Natural History 58, 65-113.

Kemp, D.B., Coe, A.L., 2007. A nonmarine record of eccentricity forcing through the Upper Triassic of southwest England and its correlation with the Newark Basin astronomically calibrated geomagnetic polarity time scale from North America. Geology 35, 991-994.

Kent, D.V., Clemmensen, L.B., 1996. Paleomagnetism and cycle stratigraphy of the Triassic Fleming Fjord and Gipsdalen formations of East Greenland. Bulletin of the Geological Society of Denmark 42, 121-136.

Kent, D.V., Malnis, P.S., Colombi, C.E., Alcober, O.A., Martínez, R.N., 2014. Age constraints on the dispersal of dinosaurs in the Late Triassic from magnetochronology of the Los Colorados Formation (Argentina). Proceedings of the National Academy of Sciences 111, 7958-7963.

Kent, D.V., Olsen, P.E., 2000. Magnetic polarity stratigraphy and paleolatitude of the Triassic–Jurassic Blomidon Formation in the Fundy basin (Canada): implications for early Mesozoic tropical climate gradients. Earth and Planetary Science Letters 179, 311-324.

Kent, D.V., Olsen, P.E., Muttoni, G., 2017. Astrochronostratigraphic Polarity Time Scale (APTS) for the Late Triassic and Early Jurassic from Continental Sediments and Correlation with Standard Marine Stages. Earth-Science Reviews.

Kent, D.V., Olsen, P.E., Rasmussen, C., Lepre, C., Mundil, R., Irmis, R.B., Gehrels, G.E., Giesler, D., Geissman, J.W., Parker, W.G., 2018. Empirical evidence for stability of the 405-kiloyear Jupiter–Venus eccentricity cycle over hundreds of millions of years. Proceedings of the National Academy of Sciences, 201800891.

Kent, D.V., Tauxe, L., 2005. Corrected Late Triassic latitudes for continents adjacent to the North Atlantic. Science 307, 240-244.

Knobbe, T.K., Schaller, M.F., 2018. A tight coupling between atmospheric pCO2 and sea-surface temperature in the Late Triassic. Geology 46, 43-46.

Kuroda, J., Hori, R.S., Suzuki, K., Gröcke, D.R., Ohkouchi, N., 2010. Marine osmium isotope record across the Triassic-Jurassic boundary from a Pacific pelagic site. Geology 38, 1095-1098.

Lallensack, J.N., Klein, H., Milàn, J., Wings, O., Mateus, O., Clemmensen, L.B., 2017. Sauropodomorph dinosaur trackways from the Fleming Fjord Formation of East Greenland: evidence for Late Triassic sauropods. Acta Palaeontologica Polonica 62, 833-843.

Maron, M., Rigo, M., Bertinelli, A., Katz, M.E., Godfrey, L., Zaffani, M., Muttoni, G., 2015. Magnetostratigraphy, biostratigraphy, and chemostratigraphy of the Pignola-Abriola section: New constraints for the Norian-Rhaetian boundary. GSA Bulletin 127, 962-974.

Minzoni, M., Lehrmann, D.J., Dezoeten, E., Enos, P., Montgomery, P., Berry, A., Qin, Y., Meiyi, Y., Ellwood, B.B., Payne, J.L., 2015. Drowning of the Triassic Yangtze Platform, South China, by tectonic subsidence into toxic deep waters of an anoxic basin. Journal of Sedimentary Research 85, 419-444.

Muttoni, G., Kent, D.V., Olsen, P.E., Di Stefano, P., Lowrie, W., Bernasconi, S.M., Hernández, F.M., 2004. Tethyan magnetostratigraphy from Pizzo Mondello (Sicily) and correlation to the Late Triassic Newark astrochronological polarity time scale. Geological Society of America Bulletin 116, 1043-1058.

Muttoni, G., Mazza, M., Mosher, D., Katz, M.E., Kent, D.V., Balini, M., 2014. A Middle–Late Triassic (Ladinian–Rhaetian) carbon and oxygen isotope record from the Tethyan Ocean. Palaeogeography, Palaeoclimatology, Palaeoecology 399, 246-259.

Nozaki, T., Nikaido, T., Onoue, T., Takaya, Y., Sato, K., Kimura, J.-I., Chang, Q., Yamashita, D., Sato, H., Suzuki, K., 2018. Triassic marine Os isotope record from a pelagic chert succession, Sakahogi section, Mino Belt, southwest Japan. Journal of Asian Earth Sciences: X, 100004.

Olsen, P.E., Kent, D.V., 1996. Milankovitch climate forcing in the tropics of Pangaea during the Late Triassic. Palaeogeography, Palaeoclimatology, Palaeoecology 122, 1-26.

Olsen, P.E., Kent, D.V., 1999. Long-period Milankovitch cycles from the Late Triassic and Early Jurassic of eastern North America and their implications for the calibration of the Early Mesozoic time-scale and the long-term behaviour of the planets. Phil. Trans. Royal Soc. Lond. Ser. A 357, 1761-1786.

Olsen, P.E., Kent, D.V., Sues, H.-D., Koeberl, C., Huber, H., Montanari, A., Rainforth, E., Fowell, S., Szajna, M.J., Hartline, B.W., 2002. Ascent of dinosaurs linked to an iridium anomaly at the Triassic-Jurassic boundary. Science 296, 1305-1307.

Olsen, P.E., Kent, D.V., Whiteside, J.H., 2011. Implications of the Newark Supergroup-based astrochronology and geomagnetic polarity time scale (Newark-APTS) for the tempo and mode of the early diversification of the Dinosauria. Earth and Environmental Science Transactions-Royal Society of Edinburgh 101, 201.

Onoue, T., Sato, H., Yamashita, D., Ikehara, M., Yasukawa, K., Fujinaga, K., Kato, Y., Matsuoka, A., 2016. Bolide impact triggered the Late Triassic extinction event in equatorial Panthalassa. Scientific reports 6, 29609.

Onoue, T., Yamashita, K., Fukuda, C., Soda, K., Tomimatsu, Y., Abate, B., Rigo, M., 2018. Sr isotope variations in the Upper Triassic succession at Pizzo Mondello, Sicily: Constraints on the timing of the Cimmerian Orogeny. Palaeogeography, Palaeoclimatology, Palaeoecology 499, 131-137.

Ramezani, J., Fastovsky, D.E., Bowring, S.A., 2014. Revised chronostratigraphy of the lower Chinle Formation strata in Arizona and New Mexico (USA): high-precision U-Pb geochronological constraints on the Late Triassic evolution of dinosaurs. American Journal of Science 314, 981-1008.

Ramezani, J., Hoke, G.D., Fastovsky, D.E., Bowring, S.A., Therrien, F., Dworkin, S.I., Atchley, S.C., Nordt, L.C., 2011. High-precision U-Pb zircon geochronology of the Late Triassic Chinle Formation, Petrified Forest National Park (Arizona, USA): Temporal constraints on the early evolution of dinosaurs. Geological Society of America Bulletin 123, 2142-2159.

Ruhl, M., Deenen, M., Abels, H., Bonis, N., Krijgsman, W., Kürschner, W., 2010. Astronomical constraints on the duration of the early Jurassic Hettangian stage and recovery rates following the end-Triassic mass extinction (St Audrie's Bay/East Quantoxhead, UK). Earth and Planetary Science Letters 295, 262-276.

Sato, H., Onoue, T., Nozaki, T., Suzuki, K., 2013. Osmium isotope evidence for a large Late Triassic impact event. Nature communications 4, 2455.

Schaller, M.F., Wright, J.D., Kent, D.V., 2011. Atmospheric pCO2 perturbations associated with the Central Atlantic magmatic province. Science 331, 1404-1409.

Schaller, M.F., Wright, J.D., Kent, D.V., 2015. A 30 Myr record of Late Triassic atmospheric pCO2 variation reflects a fundamental control of the carbon cycle by changes in continental weathering. Geological Society of America Bulletin 127, 661-671.

Schaller, M.F., Wright, J.D., Kent, D.V., Olsen, P.E., 2012. Rapid emplacement of the Central Atlantic Magmatic Province as a net sink for CO 2. Earth and Planetary Science Letters 323, 27-39.

Snedden, J., Liu, C., 2010. A compilation of Phanerozoic sea-level change, coastal onlaps and recommended sequence designations. Search and Discovery Article 40594.

Sues, H.-D., Olsen, P.E., 2015. Stratigraphic and temporal context and faunal diversity of Permian-Jurassic continental tetrapod assemblages from the Fundy rift basin, eastern Canada. Atlantic Geology 51, 139-205.

Sugiyama, K., 1997. Triassic and Lower Jurassic radiolarian biostratigraphy in the siliceous claystone and bedded chert units of the southeastern Mino Terrane, Central Japan. Bull. Mizunami Fossil Mus. 24, 79–193.

Trotter, J.A., Williams, I.S., Nicora, A., Mazza, M., Rigo, M., 2015. Long-term cycles of Triassic climate change: a new δ18O record from conodont apatite. Earth and Planetary Science Letters 415, 165-174.

Uno, K., Yamashita, D., Onoue, T., Uehara, D., 2015. Paleomagnetism of Triassic bedded chert from Japan for determining the age of an impact ejecta layer deposited on peri-equatorial latitudes of the paleo-Pacific Ocean: A preliminary analysis. Physics of the Earth and Planetary Interiors 249, 59-67.

Zhang, Z., Sun, Y., Lai, X., Joachimski, M., Wignall, P., 2017. Early Carnian conodont fauna at Yongyue, Zhenfeng area and its implication for Ladinian-Carnian subdivision in Guizhou, South China. Palaeogeography, Palaeoclimatology, Palaeoecology 486, 142-157.
